# Modified Diatomaceous Earth in Heparin Recovery from Porcine Intestinal Mucosa

**DOI:** 10.3390/molecules28247982

**Published:** 2023-12-07

**Authors:** Anushree Das, Devang P. Khambhati, Niko D. Longoria, Alireza Tabibi, Seyed Mohammad Davachi, Kayli Dimas, Yulianna Laurencin, Lesly Carmona, Pablo Zarate Avalos, Mahmood Karimi Abdolmaleki

**Affiliations:** 1 Department of Chemistry, University of Cincinnati, Cincinnati, OH 45221, USA; dasau@mail.uc.edu; 2 Department of Biology and Chemistry, Texas A&M International University, Laredo, TX 78041, USA; devang.khambhati@tamiu.edu (D.P.K.); sm.davachi@tamiu.edu (S.M.D.); kaylidimas@dusty.tamiu.edu (K.D.); leslycr1120@gmail.com (L.C.); pablozarate57@gmail.com (P.Z.A.); 3 Department of Physical and Environmental Sciences, Texas A&M University Corpus Christi, Corpus Christi, TX 78412, USA; nlongoria6@islander.tamucc.edu; 4 Department of Chemistry, Isfahan University of Technology, Isfahan 84156-83111, Iran; alirezatabibi@gmail.com

**Keywords:** diatomaceous earth, heparin, cationic diatomaceous earth, quaternary amine salt, amine modified diatomaceous earth, adsorption, viscosity, IR, heparin extraction

## Abstract

Heparin, a highly sulfated glycosaminoglycan, is a naturally occurring anticoagulant that plays a vital role in various physiological processes. The remarkable structural complexity of heparin, consisting of repeating disaccharide units, makes it a crucial molecule for the development of commercial drugs in the pharmaceutical industry. Over the past few decades, significant progress has been made in the development of cost-effective adsorbents specifically designed for the adsorption of heparin from porcine intestinal mucosa. This advancement has been driven by the need for efficient and scalable methods to extract heparin from natural sources. In this study, we investigated the use of cationic ammonium-functionalized diatomaceous earth, featuring enhanced porosity, larger surface area, and higher thermal stability, to maximize the isolated heparin recovery. Our results showed that the higher cationic density and less bulky quaternary modified diatomaceous earth (QDADE) could adsorb up to 16.3 mg·g^−1^ (31%) of heparin from the real mucosa samples. Additionally, we explored the conditions of the adsorbent surface for recovery of the heparin molecule and optimized various factors, such as temperature and pH, to optimize the heparin uptake. This is the introductory account of the implementation of modified diatomaceous earth with quaternary amines for heparin capture.

## 1. Introduction

**Scheme 1:**
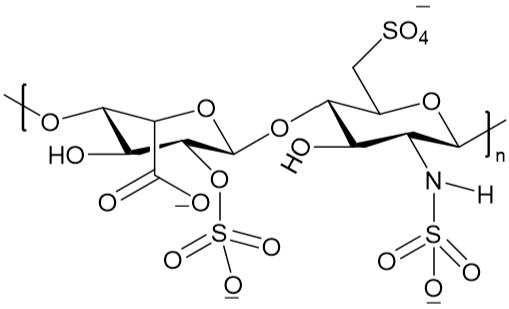
Major disaccharide unit found in heparin: (-4)-α-L-IdoA2S-(1-4)-α-D-GlcNS6S-(1-).

Heparin is a heterogeneous, sulfur-enhanced polysaccharide biopolymer consisting of alternating glucosamine and iduronic acid units with varied molecular weights (Scheme 1). The biosynthesis of heparin follows a non-template-directed fashion, leading to a significant degree of structural heterogeneity. This unique characteristic allows heparin to exhibit potent antithrombotic properties, making it an excellent anticoagulant agent. The exceptional anticoagulant properties of heparin have made it an invaluable tool in various medical applications. Its ability to selectively inhibit blood clotting has led to its widespread use in preventing and treating thromboembolic disorders, such as deep vein thrombosis, pulmonary embolism, and coronary artery disease. Additionally, heparin finds utility during surgeries, hemodialysis procedures, and extracorporeal circulation to prevent clot formation. Furthermore, research has revealed that heparin has multiple beneficial properties such as anti-angiogenesis, anti-inflammation, anti-metastasis, and anti-tumor effects. This versatility has opened avenues for exploring heparin’s potential in diverse biomedical areas, including cancer treatment, wound healing, tissue engineering, and drug delivery systems. All these applications make heparin a highly desirable pharmaceutical agent [1,2,3,4,5].

The ability to selectively remove heparin from biological fluids is an important technique in modern medicine, and ongoing investigation is concentrated on developing new compounds and techniques to advance the efficiency and selectivity of heparin adsorption. Typically, heparin is extracted from the complex biological mixture found in bovine or porcine intestinal mucosa [6]. The process begins by digesting animal organs or tissues using an alkali protease enzyme known as subtilisin, resulting in a complex mixture. Besides heparin, this digestion mixture contains nucleic acids, proteins, and other biochemicals. The next step of the heparin extraction process involves several adsorption–desorption steps [7,8,9,10,11,12]. This complex and labor-intensive process makes the extraction unviable and only very low concentrations (0.01% *w*/*w*) of heparin are isolated from enzymatically digested natural tissue [13]. To overcome these limitations, researchers and scientists have focused on designing adsorbents that can selectively capture heparin from complex mixtures, thus streamlining the extraction process while maximizing heparin yield. These economic adsorbents have been engineered using innovative materials and techniques, allowing for enhanced performance in terms of both adsorption capacity and selectivity. So far, commercially available adsorbent resin beads, such as amberlite [10], DEAE [14], Lewatti [15], and Dowex [8], are employed to obtain heparin from animal tissue. Although these adsorbents have some advantages, they have low heparin selectivity and higher costs. Therefore, it is highly desirable to conduct research to develop new adsorbents. Adsorbent surface area, porosity, surface chemistry/properties, and chemical stability, along with thermodynamic stability, are some of the crucial parameters for the maximum extraction of heparin [11,16].

To create an innovative, economically efficient, as well as chemically and thermally resistant material, we have utilized porous diatomaceous earth (DE) as the base component, which has been functionalized for the purpose of extracting heparin. DE is a naturally occurring mineral composed of fossilized diatoms, which are single-celled algae belonging to the class of Bacillariophyceae of Phylum Bacilloriophyta [17]. DE is predominantly composed of amorphous silicon dioxide (86–94%) with some alumina (Al_2_O_3_) and ferric oxide (Fe_2_O_3_) [18]. DE is an inexpensive and widely available material with significant chemical as well as thermal stability. DE is also a highly porous material with low density, high surface area, and surface roughness [19,20,21]. There are hydroxy groups on the surface of DE particles, which can be utilized to functionalize DE with various functional groups/chains. These properties make DE an ideal support material for adsorbents, such as metal adsorbents [22,23,24], toxin adsorbents [25,26,27], superhydrophobic coatings/materials [28,29], oil-spill adsorbents from water bodies [30], and also in drug delivery [31,32,33,34].

**Scheme 2:**
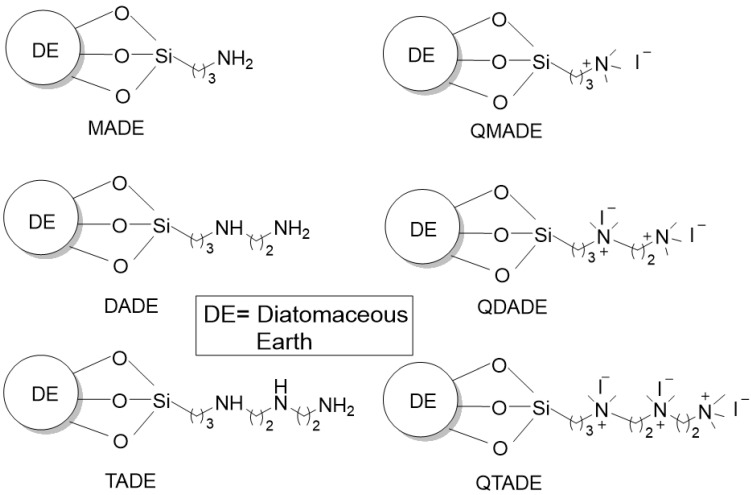
Amine-functionalized diatomaceous earth adsorbents.

Heparin is traditionally extracted from the intestinal mucosa of pigs using ion exchange adsorption technology [35]. Typically, a positively charged polymer or biomolecule binds with the negative sulfate and carboxylate sites of heparin via electrostatic interactions (Scheme 1) [36,37,38,39]. The extraction of heparin using this method has been widely utilized in various applications [35,37,40,41], such as an FDA-approved antidote for heparin to substitute protamine, for the production of label-free sensors for heparin monitoring on molecularly imprinted resin, and to regulate bacterial activity [38,39,42,43,44,45,46]. Therefore, in this research, we have functionalized DE with quaternary ammonium functional groups containing side chains (Scheme 2) and utilized them for heparin extraction from the porcine intestinal mucosa. To the utmost of our awareness, this is the initial account of heparin adsorption using functionalized DE materials.

We optimized heparin adsorption by modulating several variables including pH, dosage of the adsorbent, interaction time, and process temperature, and demonstrated that the greater positive charge strength and more compact environment (e.g., QDADE) are optimal for a greater heparin recovery. QDADE can be reused after it has been washed with a brine solution and it will remain stable during the heparin recovery process, even after five cycles, making it a commercially practical product. To ascertain QDADE’s anticoagulant capability in comparison to the commercial amberlite FPA98 Cl, potency measurements were taken using sheep’s plasma. Based on these studies, quaternary ammonium-functionalized DE has great potential for heparin uptake.

## 2. Results and Discussion

### 2.1. Preparation and IR Identification of Quaternary Ammonium-Functionalized Diatomaceous Earth

**Figure 1:**
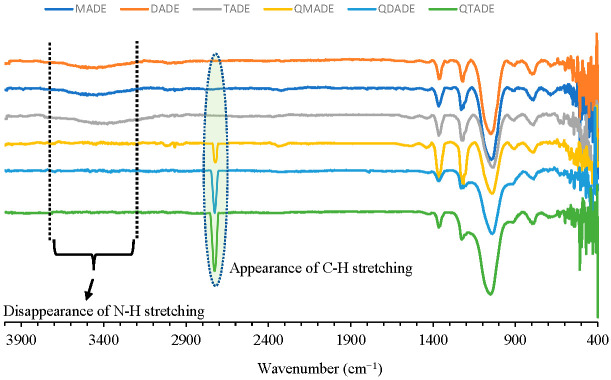
FTIR Spectra of DE derivatives and their quaternary salts (MADE, DADE, TADE, QMADE, QDADE, and QTADE).

To synthesize amine-functionalized DE (MADE, DADE, and DADE), DE was reacted with various silanes in the presence of p-toluene sulfonic acid (PTSA). QMADE, QDADE, and QTADE were synthesized by combining methyl iodide with the respective amine-functionalized DE in excess amounts, and the final products were authenticated using Infrared (IR) spectra. The IR studies of amine-modified DE and its salts are illustrated in Figure 1. The bands at 1224, 1049, and 788 cm^−1^ are for Si-CH stretching and δ Si-O-Si and Si-O-Si stretching, respectively. The bending of C-H (alkane) can be seen at 1365 cm^−1^. Additional vibrational frequencies of both asymmetric and symmetric C-H stretching can be observed in the quaternary salts situated between 3014 and 2690 cm^−1^. The N-H stretching frequencies, present in various DE amides (such as at 3473 cm^−1^ in MADE), disappeared in quaternized salts [46,47,48,49].

### 2.2. Heparin Adsorption Studies

The parameters of pH, contact time, dosage, and temperature were adjusted to design an adsorption technique for the recovery of heparin from porcine mucosa, which contains many other biomolecules and enzymes. This process includes the adsorption of heparin on an adsorbent at biological conditions, followed by the desorption of the previously captured molecules to yield pure heparin. Initial adhesion studies were designed in pure heparin solutions. All experiments were conducted in three sets, and the most efficient adsorbent was ascertained based on its optimal affinity in a pure heparin solution. We further investigated the adsorbent’s reusability to understand its commercial practicality. Kinetic and thermodynamic analyses were conducted to study the adsorption mechanism. An aqueous solution of 1000 mg L^−1^ heparin was typically prepared, and 0.5 g of quaternary ammonium-modified DE was added to the solution and agitated for 5 h at 65 °C in an incubator. The adsorbed heparin amount was then measured using the methylene blue method [40,50]. In aqueous solutions, QDADE salt adsorbs heparin 2.7-fold more than QMADE salt, as shown in Table 1. It also had the capacity to take in 1.7 times more heparin than QTADE. The longer alkane chain leads to increased hydrophobicity and steric hindrance in QTADE. Thus, although QTADE has the highest cation concentration, its heparin adsorption is lower compared to QDADE. Consequently, QDADE was selected to further analyze heparin adhesion in the real samples taken from the intestinal mucosa of pigs which is described in Section 2.2.1, Section 2.2.2, Section 2.2.3, Section 2.2.4, Section 2.2.5 and Section 2.2.6.

**Figure 2:**
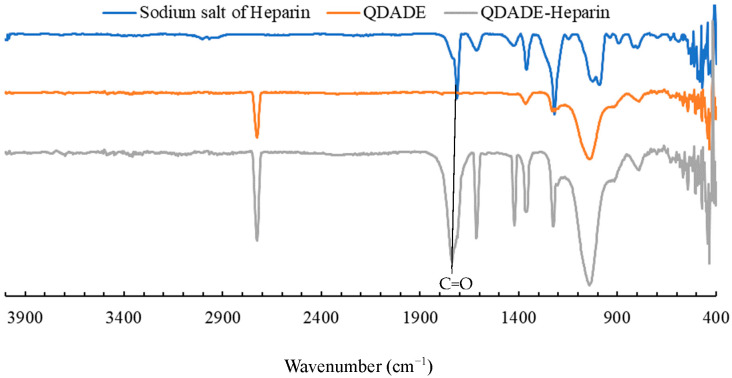
The IR spectra of the commercial heparin sodium salt, QDADE, and pre-adsorbed standard heparin on the QDADE.

Pure heparin adsorption from an aqueous solution on the QDADE surface was further studied by Fourier-Transformed IR analysis (FTIR, Figure 2). The FTIR spectra of the commercial sodium salt of heparin and QDADE were compared and the C=O stretching frequency at 1712 cm^−1^ in QDADE–heparin conjugate confirms that the heparin adsorbed onto the QDADE surface [48,49,51].

**Figure 3:**
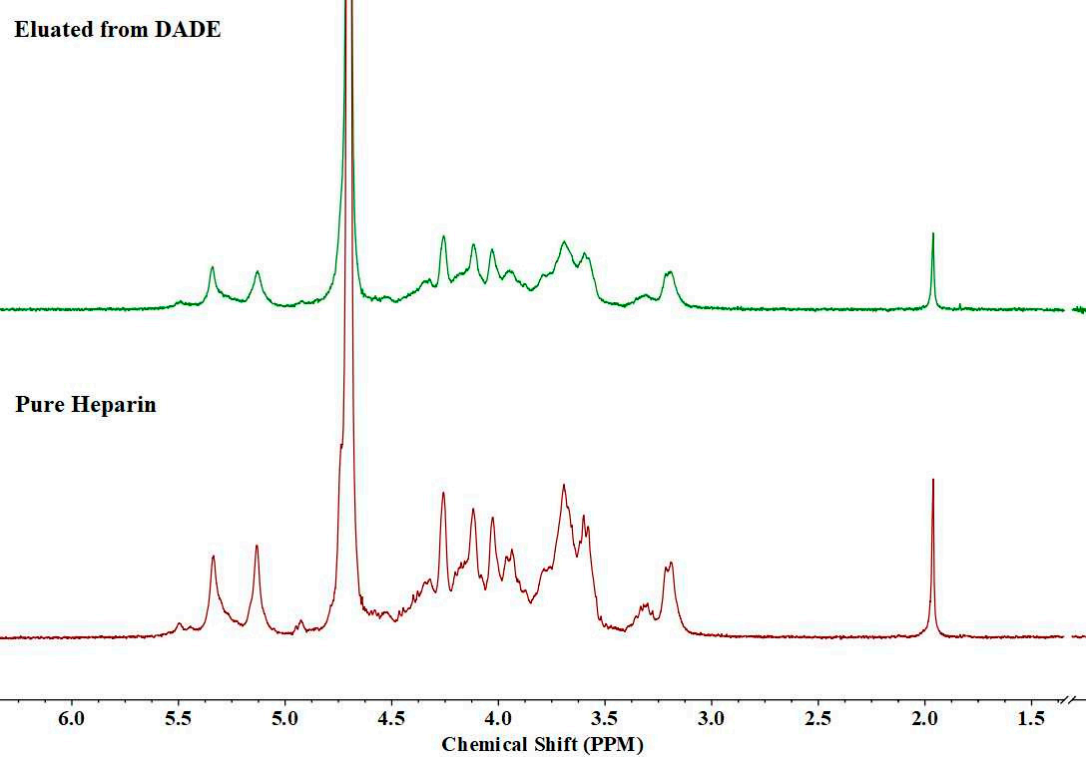
^1^H NMR spectra (400 MHz, D_2_O) of pure heparin (red) and real heparin (green) which is adsorbed and extracted from QDADE.

NMR studies were performed to analyze and compare the heparin extracted from QDADE with commercial heparin. Figure 3 demonstrates that the peaks observed in the pure heparin eluted from QDADE closely align with those found in commercial heparin. For example, the methyl peaks in the N-acetyl glucosamine region (GlcNAc) of both the isolated and commercial heparin are located at 1.96 ppm (Figure 3) [50]. This indicates that QDADE material has selectively adsorbed the heparin from porcine mucosa.

#### 2.2.1. Molecular Weight Calculations Using Viscosity

To obtain the intrinsic viscosity of the heparin ([η]) using the Kraemer equation, heparin solutions with various concentrations of 0.05–0.3 g/mL in DI water were prepared. The intrinsic viscosity of heparin is approximately 0.1561 dL/g and the molecular weight using the Mark–Houwink–Sakurada equation was calculated to be 15,750 g/mol. This value is close to the molecular weight of commercial heparin, which is around 15,500 g/mol.

#### 2.2.2. pH Optimization

**Figure 4:**
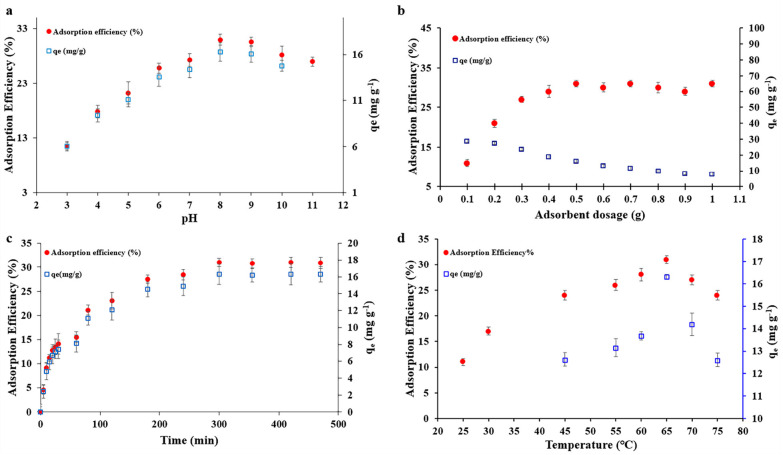
Adsorption efficiency of heparin over QDADE by optimizing (**a**) pH (protocol: 0.5 g of QDADE in 20 mL of 1315 mg L^−1^ mucosa sample at 65 °C for 300 min of contact time), (**b**) adsorbent dosage (conditions: 25 mL of 1315 mg L^−1^ mucosa sample at pH = 8, at 65 °C over 300 min), (**c**) contact time (conditions: 0.5 g of QDADE in 25 mL of 1315 mg L^−1^ real mucosa sample at pH = 8, and at 65 °C), and (**d**) temperature (conditions: 0.5 g of QDADE in 25 mL of 1315 mg L^−1^ real sample at pH = 8 for 300 min).

The pH of the medium greatly influences the adsorption properties of quaternized DE. The intestinal mucosa from pigs as the real biological sample was used to conduct experiments and optimize Heparin’s adsorption rate by QDADE through pH modulation of the real sample solution between 3 and 11. ELISA kit assay and/or a sheep plasma test were used to measure the amount of heparin adsorbed by QDADE. The heparin adsorption increases with a pH between three and eight (Figure 4a). At pH = 8, the adsorption was found to be the highest with 31% efficiency and 16.3 mg·g^−1^ capacity, which slightly decreased under a higher pH. This is due to the fact that heparin is deprotonated, causing it to become more negatively charged and, therefore, more accessible to the positively charged sites of QDADE. It was determined that this was the optimum pH for heparin uptake as QDADE neutralizes at a higher pH, resulting in a reduced absorption.

#### 2.2.3. Optimization of the Adsorption Dosage

We studied the impact of the adsorbent dosage on heparin consumption in a real porcine mucosa sample by varying the QDADE concentrations from 100 to 1000 mg. The adsorption capacity amplified to 28.9 mg·g^−1^ when the QDADE dosage was upped to a maximum of 100 mg (Figure 4b). At a 500 mg dosage, the adsorption efficiency exhibited a peak of 31%. As the adsorbent dosage was raised, the adsorption rate stayed nearly unchanged, though the adsorption capacity dropped. The initial adsorption surge was due to the greater surface area containing active sites on the QDADE surface. Real heparin mucosa samples contain a variety of extra components, such as dermatan and chondroitin sulfate, which may interfere with heparin uptake by bonding to the surface of the adsorbent [50]. This could decrease the adsorption ability at higher dosages. Furthermore, a decrease in capacity (28.9 to 8.15 mg·g^−1^) was noticed due to the augmentation of mass (m) in Equation (7).

#### 2.2.4. Temperature and Duration of Adsorption Effect on Heparin Uptake in Real Sample

The adhesion of heparin on QDADE surfaces increases over time as the negatively charged heparin diffuses from the solution to adhere to the QDADE surfaces. After a 300 min contact period, the adsorption efficiency (31%) and capacity (16.3 mg·g^−1^) of heparin on QDADE surfaces experienced a significant rise. This is evidenced by the data in Figure 4c. The adsorption was found to be quite steady after a point, suggesting that the QDADE had reached saturation in the experimental settings. The temperature of the solution had a significant effect on the adsorption affinity. Thus, a few experiments were conducted with the temperature varying from 25 °C to 75 °C. The results (Figure 4d) show that when the temperature was increased to 65 °C, the adsorption efficiency and capacity reached their highest values of 31% and 16.3 mg·g^−1^, respectively. The decreased solution viscosity at elevated temperatures enabled the heparin to pass more quickly in the solution, leading to efficient interactions with the QDADE surface. A drop beyond 65 °C may be attributed to the decomposition of the heparin molecules at higher temperatures.

#### 2.2.5. Kinetic and Thermodynamic Studies

##### Kinetic Study

**Figure 5:**
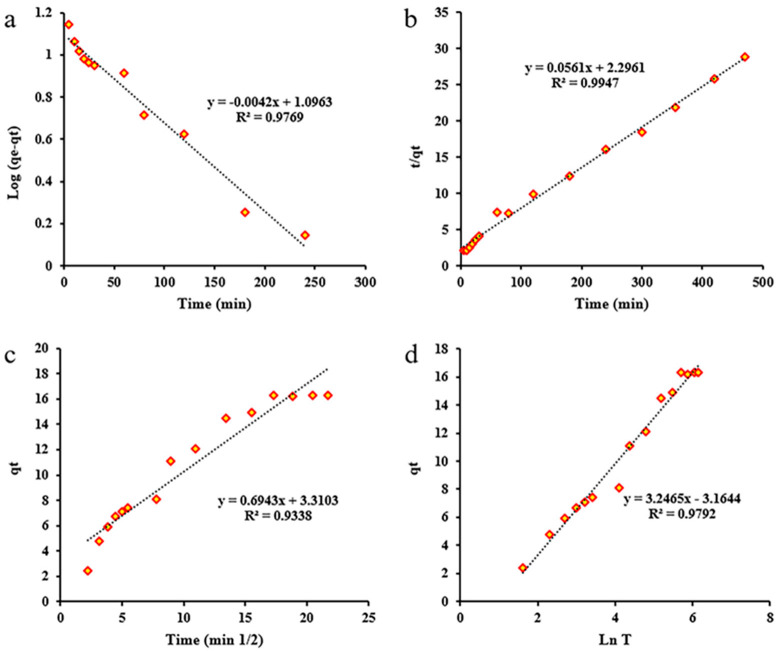
The pseudo-first order (**a**), pseudo-second order (**b**), intraparticle diffusion (**c**), and Elovich (**d**) kinetic models for heparin adsorption by QDADE in the optimal conditions.

The kinetic studies examine the speed of the chemical process in order to find the effect of each factor and also the type of reaction mechanism between species. The kinetics of chemical reactions are dependent on the concentration of substrate and the influence of the species in the reaction. Some mathematical models were developed to investigate the adsorption kinetic such as pseudo-firs order, pseudo-second order, Elovich, and intraparticle diffusion. These models were evaluated to find the adsorption mechanism of heparin molecules on the QDADE (Figure 5). The linear form of models, related coefficients, and constants obtained from the equations are listed in Table 2.

According to the obtained results, by comparing the correlation coefficients models, the pseudo-second-order model with a linear coefficient of 0.9947 was presented as the best agreement to the adsorption mechanism. The appropriate matching of the obtained experimental adsorption capacity with the calculated adsorption capacity in the pseudo-second-order model is another confirmation that the adsorption kinetic more likely to follow this model [52,53,54,55,56]. The adherence of kinetic from the pseudo-second-order model shows that the chemical adsorption has a more effective role in the adsorption of heparin by the QDADE.

**Table 2:** The linear form and calculated parameters of kinetic models for heparin adsorption on QDADE [57,58].

Model	Linear Equation	Parameter	Value
Pseudo-first order	Ln(q_e_ − q_t_) = Lnq_e_ − k_1_ × t	K_1_	0.0096726
		q_e (cal)_	12.482
		R^2^	0.9769
Pseudo-second order	t/q_e_ = 1/(k_2_ × q_e_^2^) + (1/q_e_) × t	K_2_	0.00137
		q_e (cal)_	17.825
		R^2^	0.9947
Intraparticle Diffusion	q_t =_ k_i_ × √t + x_i_	K_diff_	0.6943
		C	3.3103
		R^2^	0.9338
Elovich	Log(C_0_/(C_o_-q_t_ × m)) = Log(k_0_/2.303 × V) + αLogt	β	0.30802
		α	1.224
		R^2^	0.9792
		q_e (exp)_	16.3

##### Thermodynamic Study

Thermodynamic parameters of heparin adsorption by QDADE include changes in standard Gibbs free energy (∆*G*°), changes in standard enthalpy (∆*H*°), and changes in standard entropy (∆*S*°) can be calculated using the following equations:

(1) $$LnK_{c}=-\frac{∆H{}^{\circ}}{RT}+\frac{∆S{}^{\circ}}{R}$$

In the above equations, *K_c_* is the equilibrium constant (mL·g^−1^), which is obtained from the following equation:

(2) $$K_{c}=\frac{\left(C_{0}-C_{e}\right)}{C_{e}}\times \frac{V}{M}$$

**Figure 6:**
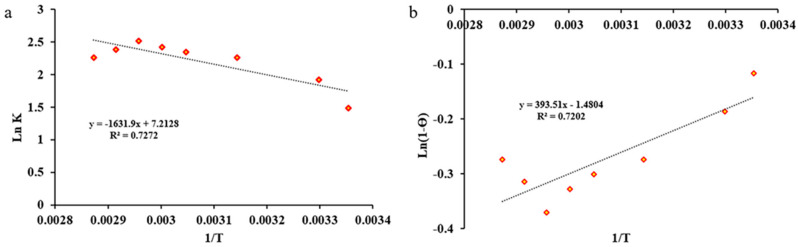
The van’t Hoff plot (**a**) and ln(1 − *θ*) versus 1/*T* plot (**b**) of heparin adsorption on QDADE absorbent.

By drawing the van’t Hoff diagram, (ln*K_c_* versus 1/*T*) (Figure 6), the values of changes in the enthalpy and entropy can be calculated from the slope and intercept, respectively. The standard Gibbs free energy changes at the desired temperatures are determined in Equation (3).

(3) ∆G=∆H−T∆S

The activation energy value (*E_a_*) and the sticking probability (S*) were calculated from the slope and intercept of ln(1 − *θ*) versus 1/*T* plot, according to Equations (4) and (5) [59,60,61,62].

(4) $$Ln\left(1-\theta \right)=LnS^{*}+\frac{E_{a}}{RT}$$

(5) $$\theta =\left[1-\frac{C_{e}}{C_{0}}\right]$$

The thermodynamic variables were extracted at different temperatures and the obtained results are collected in Table 3. The negative value of the standard Gibbs free energy changes indicates the spontaneity of the adsorption process. In the following, it should be noted that the positive standard enthalpy changes of the reaction are a sign of the endothermic nature of the absorption process. On the other hand, the positivity of the standard entropy changes of the system indicates the increase of disorder in the interface of the solid/solution absorption process. The positive values of *E_a_* indicate that the adsorption process is endothermic in nature. Also, the value of S* equal to 0.227 indicates the proper stick of heparin molecules on the surface of the adsorbent [59,60,61,62].

#### 2.2.6. Sorbet Reusability

**Figure 7:**
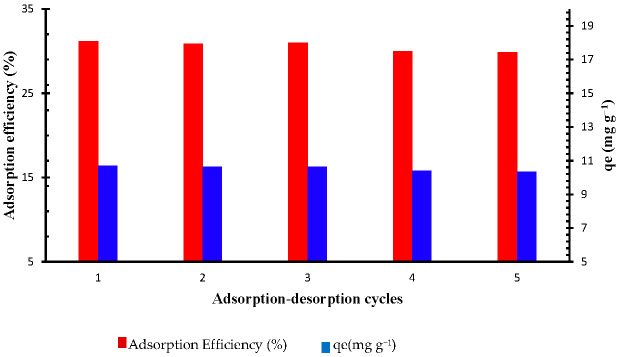
Stability test of QDADE over five adsorption–desorption heparin recovery cycles.

QDADE surpassed other silica-based adsorbents in its ability to adsorb heparin from biological samples, displaying a superior performance [11]. Specifically, it achieved an adsorption efficiency of approximately 31% and a capacity of 16.4 mg·g^−1^ under the optimized conditions. To test the commercial viability of the material, we subjected QDADE to five adsorption–desorption cycles with a harsh regeneration condition of washing with a saturated sodium chloride solution at 55 °C for 3 h, followed by a Milli-Q water clean [41,63] The results (Figure 7) demonstrate that QDADE is a highly effective adsorbent that exhibits remarkable stability and can be used multiple times, even after undergoing five cycles of adsorption–desorption with a harsh regeneration process.

#### 2.2.7. Sheep Plasma Clotting Assay

In order to assess the purity of heparin acquired through the utilization of QDADE, we conducted a sheep plasma clotting assay under optimized conditions to quantify its anticoagulant efficacy. Additionally, we performed an examination of heparin obtained using amberlite FPA98 Cl resin, following a previously documented procedure [40]. Our findings indicate that the anticoagulant potency of heparin obtained from QDADE (49 ± 2.1 U per gram of mucosa) closely mirrors that obtained from the commercial amberlite FPA98 Cl resin (50 ± 2.4 U per gram of mucosa).

## 3. Experimental

DE (natural diatomaceous earth, DIAFIL 525) was purchased from Imerys, USA. (3-Aminopropyl)triethoxysilane, *N*-[3-(Trimethoxysilyl)propyl]ethylenediamine,3-[2-(2-Aminoethylamino)ethylamino]propyl-trimethoxysilane, p-Toluene sulfonic acid (PTSA) monohydrate, methyl iodide, toluene, and ethanol were purchased from Fisher Scientific, USA. We acquired heparin sodium salt (analytical grade), sheep plasma, calcium chloride, hydrochloric acid (37%), sodium hydroxide, methanol, and ethanol from VWR, USA. All chemicals were consumed as received. Sample preparation for the adsorption experiments was performed using Milli-Q water. (Sigma Aldrich, Saint Louis, MO, USA). Porcine intestinal mucosa containing around 1300 mg/L heparin was locally sourced. The subtilisin enzyme was obtained from STERM Company, USA. Commercial amberlite FPA98 Cl resin was sourced from Dow Chemical, USA. MyBiosource, San Diego, CA, USA supplied the heparin ELISA kits.

The SPECTROstar nano microplate reader (BMG LABTECH) was employed to calculate the concentration of heparin in both pure and real samples, while the Shimadzu IRAffi FT-IR instrument was utilized to characterize the amine-functionalized diatomaceous earth and heparin sodium salt.

### 3.1. Synthesis

#### 3.1.1. Synthesis of Amine-Functionalized DE [29,64]

DE (1 g) was stirred at room temperature in toluene (15 mL) for 30 min in three separate vials. PTSA monohydrate (~0.02 g) and different silanes [(3-Aminopropyl)triethoxysilane (1.25 g), *N*-[3-(Trimethoxysilyl)propyl]ethylenediamine (1.5 g), 3-[2-(2-Aminoethylamino)ethylamino]propyl-trimethoxysilane (2 g)] were added separately in the vials to produce MADE, DADE, and TADE, respectively. The reaction mixtures were stirred at 50 °C for 4 h and cooled down to room temperature. The slurries were filtered and washed with toluene to obtain amine-functionalized DE particles, which were kept under reduced pressure in a desiccator. MADE, DADE, and TADE (1.52 g, 1.71 g, and 1.87 g, respectively) were recovered as powders.

#### 3.1.2. Methyl Iodide Treatment of Amine-Functionalized DEs [11,37]

Different volumes of methyl iodide (1.0, 1.5, and 2.0 mL) were added individually to the Milli-Q water solutions (12.5 mL) of MADE, DADE, and TADE (1 g per material), respectively, at pH ~6.0. An excessive amount of methyl iodide (CH_3_I) was employed for the synthesis. The reaction mixtures were stirred at room temperature for 4 h and then strained. The residues were washed with ethanol, dried under reduced pressure, and examined with FTIR. QMADE, QDADE, and QTADE (1.31 g, 1.47 g, and 1.53 g, respectively) were acquired as powders, placed under cold nitrogen, and used for various adhesion studies.

#### 3.1.3. Sample Preparation for IR Measurements

To eliminate any remaining solvents and moisture, functionalized DE was first subjected to a vacuum oven both before and after heparin adsorption at temperatures of 60 °C and 25 °C, respectively. Following this, the materials were converted into consistent powders using a mortar and pestle in order to conduct IR experiments.

#### 3.1.4. Viscosity Measurement

To determine the intrinsic viscosity of the heparin ($\left[\eta \right]$), we conducted measurements in a dilute deionized water (DI) solution at 40 °C. This was carried out using a Minivisc 3000 series instrument (Spectro Scientific, Chelmsford, MA, USA), following our established methodology of measuring relative viscosity (ηrel=ηsampleηDI water), and applying the Kraemer equation ($\frac{\mathrm{Ln}\eta_{\mathrm{rel}}}{C}=\left[\eta \right]-\;K^{’}{\left[\eta \right]}^{2}C$). By plotting LnηrelC against the concentration (C), we obtained the necessary data [11,65]. Finally, the molecular weight (Mw) of the heparin was calculated using the Mark–Houwink–Sakurada equation ($\left[\eta \right]=K.M_{w}^{\alpha }$). In this equation K and α are constants for heparin in water at 40 °C that have been reported to be 3.16 × 10^−5^ and 0.88, respectively [11,65].

### 3.2. Solutions and Methods

A 1000 ppm heparin stock solution was created in Milli-Q water, and the plasma of sheep and ELISA heparin kits were used to assess the efficacy and capacity of heparin adsorption by utilizing Equations (6) and (7) in the intestinal mucosa of pigs. This process is documented in a prior article [50].

(6) $$Adsorption\;efficiency\;(\%)=\frac{\left(C_{0}-C_{e}\right)}{C_{0}}\times 100$$

(7) $$Adsorption\;capacity\;(q_{e})=\frac{\left(C_{0}-C_{e}\right)}{m}\times V$$

where *C*_0_ and *C*_e_ (mg L^–1^) are the initial and equilibrium heparin concentrations (measured with the ELISA kit), *V* (L) is the volume of the mucosa solution, used for heparin adsorption, and *m* is the mass of the adsorbent used (g).

To assess the adsorption capabilities of the DE materials, we conducted the following procedure: Initially, 250 mg of the quaternary salts of DE were added to the 25 mL aqueous solutions containing 1000 ppm of heparin sodium. The resulting solutions were placed in an incubator and stirred for 3 h at a temperature of 55 °C. Subsequently, 5 mL of each solution was withdrawn and filtered using a syringe filter, enabling the separation of the supernatant. To measure the concentration of heparin in the supernatant, we employed the methylene-blue-assisted spectrophotometric method. Methylene blue (MB), a cationic metachromatic dye, was utilized due to its specific affinity towards heparin. In this methodology, 1 mL of the heparin solution was mixed with 1 mL of the MB solution (10 mg L^−1^). After vigorous mixing using a vortex mixer for 10 min, the absorbance of the resulting mixture was measured using a plate reader. The intensity of the band observed at λ_max_ = 663 nm, corresponding to unbound MB, was recorded. MB forms dimers with heparin, which decreases the concentration of free MB, resulting in a decreased intensity at 663 nm. A pre-plotted standard calibration curve was used to calculate the concentration of heparin after adsorption [12,40,50].

## 4. Conclusions

In this study, we explored the possibility of recovering heparin using quaternary ammonium-functionalized diatomaceous earth (DE). As far as we know, this is the first instance of positively charged DE being used for heparin recovery. We prepared a number of quaternary ammonium-modified diatomaceous earth and discovered that QDADE was the most effective for heparin recovery, with an adsorption rate of up to 31% from the actual sample. The effective performance of QDADE can be attributed to multiple factors, including its high charge density, reduced hydrophobicity, and the presence of less steric crowding in the vicinity of the adsorption sites of QDADE. The higher charge density ensures stronger electrostatic interactions between QDADE and heparin molecules, facilitating efficiency. The adsorption process was found to follow the pseudo-second-order kinetic model and to be thermodynamically endothermic and feasible. Furthermore, further research on optimizing heparin adhesion was studied by adjusting parameters such as temperature, pH, and dosage.

## Figures and Tables

**Table 1:** Adsorption capacities and efficiencies of amine-modified DE and their quaternary salts when exposed to a pure heparin standard solution.

Compound	Adsorption Efficiency (%)	Adsorption Capacity (mg·g^−1^)
DE	below 5	below 3
MADE	below 5	below 3
DADE	below 5	below 3
TADE	below 5	below 3
QMADE	23	12.1
QDADE	62	32.6
QTADE	36	19

**Table 3:** The value of thermodynamic parameters for heparin adsorption on the QDADE.

∆*H*° (kJ/mol.K)	∆*S*° (kJ/mol)	S*	*E_a_* (kJ/mol)	Temperature (K)							
				298.15	303.15	318.15	328.15	333.15	338.15	343.15	348.15
13.56	59.96	0.2275	3.271	∆*G* (KJ/mol)							
				−17.86	−18.16	−19.06	−19.66	−19.96	−20.26	−20.56	−20.86

## Data Availability

Data are contained within the article.

## Abbreviations

DE	Diatomaceous earth
MADE	3-Aminopropyl-functionalized DE
DADE	3-(Ethylenediamino)propyl-functionalized DE
TADE	3-(Diethylenetriamino)propyl-functionalized DE
QMADE	Quaternarized MADE
QDADE	Quaternarized DADE
QTADE	Quaternarized TADE
